# British torture in the ‘war on terror’

**DOI:** 10.1177/1354066116653455

**Published:** 2016-06-16

**Authors:** Ruth Blakeley, Sam Raphael

**Affiliations:** University of Kent, UK; University of Westminster, UK

**Keywords:** Human rights, imperialism, prisoner abuse, torture, UK, war on terror

## Abstract

Despite long-standing allegations of UK involvement in prisoner abuse during counterterrorism operations as part of the US-led ‘war on terror’, a consistent narrative emanating from British government officials is that Britain neither uses, condones nor facilitates torture or other cruel, inhuman or degrading treatment and punishment. We argue that such denials are untenable. We have established beyond reasonable doubt that Britain has been deeply involved in post-9/11 prisoner abuse, and we can now provide the most detailed account to date of the depth of this involvement. We argue that it is possible to identify a peculiarly British approach to torture in the ‘war on terror’, which is particularly well-suited to sustaining a narrative of denial. To explain the nature of UK involvement, we argue that it can be best understood within the context of how law and sovereign power have come to operate during the ‘war on terror’. We turn here to the work of Judith Butler, and explore the role of Britain as a ‘petty sovereign’, operating under the state of exception established by the US executive. UK authorities have not themselves suspended the rule of law so overtly; indeed, they have repeatedly insisted on their commitment to it. Nevertheless, they have been able to construct a rhetorical, legal and policy ‘scaffold’ that has enabled them to demonstrate at least procedural adherence to human rights norms while, at the same time, allowing UK officials to acquiesce in the arbitrary exercise of sovereignty over individuals who are denied any access to appropriate representation or redress in compliance with the rule of law.

## Introduction

In October 2015, after 14 years’ detention without trial at Guantánamo Bay and repeated torture by the US military, UK resident Shaker Aamer was finally freed and returned to Britain. Almost immediately, his lawyers signalled that he would sue the British government over its involvement in his abuse. Specifically, Aamer alleges that UK security and intelligence officials were aware of his mistreatment at the hands of the Americans, and were on one occasion present (Davies et al., 2015a). In an earlier set of legal proceedings, Aamer had also alleged that British intelligence supplied ‘knowingly false information’ to US interrogators, which led to his initial capture, detention and rendition from Afghanistan to Guantánamo Bay (Swann, 2012). In return, the UK government has consistently denied any involvement in, or responsibility for, Aamer’s abuse, stating on his release that it ‘did not accept allegations of … complicity in his mistreatment’ (Davies et al., 2015b). Statements by UK officials regarding his case have always emphasised Britain’s commitment to human rights and the rule of law, and stressed the repeated representations made to the US government to secure his release.

Although the time spent by Aamer in arbitrary detention surpasses all other British citizens and residents known to have been caught up in the ‘war on terror’, neither the nature of Aamer’s allegations nor the denials by the UK government in response are particularly new. There have been literally hundreds of allegations concerning UK involvement in prisoner abuse since September 2001, both within and outside the military theatres in Iraq and Afghanistan. These cases have involved allegations of direct participation in abuse by British personnel, as well as the provision of indirect support for abuses carried out by foreign government agents (Gibson, 2013: 7). In response, the UK government has consistently and categorically denied involvement in torture and other prisoner mistreatment, while simultaneously working to block relevant information from being released into the public sphere. This position has been sustained even as the government has established, or engaged with, a number of parliamentary, police and judicial investigations into allegations of British involvement.

In this article, we examine the allegations of involvement by the British intelligence and security services in torture and other prisoner mistreatment, and the consistent strat-egies of denial that the UK government has pursued in response. We also argue that such denials are untenable. Our argument is underpinned by careful documentary analysis of dozens of declassified documents, court proceedings, victim testimonies and investigative reports by a number of leading human rights organisations, as well as detailed analysis of flight data relating to specific prisoner renditions. Our work with The Rendition Project — an academic project at the forefront of investigations into the use of torture as part of the ‘war on terror’ — has allowed us to establish beyond reasonable doubt that Britain has been deeply and directly involved in post-9/11 prisoner abuse. Furthermore, while many details remain unknown, we are now in a position to provide the first detailed academic analysis of the depth and scope of this involvement.

Understanding the scope of British involvement in torture since 9/11 forms one aspect of the research puzzle at the heart of this article. In addition, we want to understand the characteristics of this involvement, and to account for these. In this light, we argue here that it is possible to identify a peculiarly British approach to torture in the ‘war on terror’, one that is particularly well-suited to sustaining a narrative of denial. Of course, there is a long history of the involvement of the British state in prisoner abuse, particularly as a tool of colonial control in the expansion, maintenance and drawdown of the British Empire (e.g. see the excellent work on the systematic use of torture by the colonial administration in Kenya (Anderson, 2005; Elkins, 2005)). However, while acknowledging some similarities between such colonial-era torture and the ways in which Britain has been involved in prisoner abuse in the ‘war on terror’, we argue that the nature of British involvement has changed in many respects, and that these changes need to be understood within the context of the changing nature of broader imperial relations. Thus, at the height of British colonialism, torture and human rights violations took place against peoples and in territories under direct British rule, even if that rule was deeply contested. As a tool of formal imperial policing throughout the Empire, torture and other abuse was carried out by both British military and security officials directly, and by local colonial agents. Depending on the scale of local resistance to British control, the use of torture was targeted against either narrow groups of suspected insurgents or supposedly supportive populations *en masse*. UK involvement in torture during the ‘war on terror’ looked very different, and was reflective of both Britain’s secondary role in the US neo-imperial project and a clear desire to retain a public commitment to liberal norms in the fight against terror. As we will show, Britain has been able to benefit from the US suspension of core commitments under international law, while appearing to maintain a commitment to human rights obligations. Specifically, it has developed a particular set of constraints — a rhetorical, legal and procedural ‘scaffold’ within which British officials have had to operate — which, in turn, have shaped contemporary involvement in torture in new ways.

Ultimately, British intelligence and security agencies have had to ensure that any substantive involvement in prisoner abuse takes place in ways that maintain a level of *procedural* adherence to human rights norms and legal commitments, thus enabling ministers to routinely proclaim the continued and unwavering prioritisation of human rights in the fight against terror. This approach has been markedly different from that of the US, which suspended core commitments under international law and developed specific politico-legal justifications for the indefinite detention and torture of ‘terror suspects’ (for more on the ‘state of exception’ thesis in the ‘war on terror’, see, among many, Agamben, 2005; Butler, 2004; Huysmans, 2006; Jabri, 2006; Neal, 2009). However, these two approaches are not unconnected, and we argue here that it was the US decision to reinterpret its commitments under international law, and subsequently to develop a set of detention and interrogation programmes that sat outside the law, which enabled the UK to engage in prisoner abuse while simultaneously maintaining a public appearance of adherence to existing international human rights law.

We argue that with the creation of a space unhindered by international law comes a new exercise of sovereign power. Contemporary British involvement in torture has taken the form it has because of the ways in which the ongoing development of neoliberal biopolitical power entwines with still-existing sovereign power. The interaction of these two forms of power render bureaucracy and procedure central to the use (and disguise) of British torture. Sovereign power is exercised by administrative bureaucracies, in which unelected officials, described by Judith Butler (2004: 51–66) as ‘petty sovereigns’, decide who will be imprisoned arbitrarily and indefinitely. Our argument is that Britain was able to fully exploit the public extension of sovereign power by the US government, allowing unelected officials within the UK security and intelligence services huge latitude in the abuse and torture of prisoners, while, at the same time, maintaining a public commitment to existing legal obligations. We show, therefore, that the declaration of a state of exception in its national and international forms by one state can open up the way for the unaccountable exercise of sovereign power by other states.

We begin by outlining the allegations made about British complicity in prisoner abuse, and show how a narrative of denial emerged and was maintained by British officials. We then account for the particular approach adopted by British intelligence and security agencies which ensured that the narrative of denial was more likely to be sustained. We then go on to show the extent of British complicity in prisoner abuse, and provide the most comprehensive and detailed account of this to date. We close with some reflections on how the new exercise of sovereign power might be challenged to ensure that the law is upheld to protect all citizens from arbitrary and indefinite detention and torture.

## Allegations and denials of British torture

There have been numerous allegations of British torture in the ‘war on terror’. Testimony from former prisoners, security personnel speaking off the record to journalists and dogged investigative work by a number of journalists, lawyers and non-governmental organisations (NGOs) have brought many details to light. As a result, the incoming Coalition government in 2010 launched a judge-led inquiry to examine ‘whether Britain was implicated in the improper treatment of detainees, held by other countries, that may have occurred in the aftermath of 9/11’ (Cameron, 2010a). Although this was closed down before it had had the chance to call witnesses, the Detainee Inquiry compiled over 20,000 documents from the UK intelligence agencies and government departments, and issued a preliminary report which made clear that these documents pointed to UK involvement in prisoner mistreatment. The Inquiry identified 40 cases that ‘best illustrate the key issues it was asked to investigate’, and ‘prepared detailed analyses, which are necessarily confidential, of documents received by the Inquiry on each of the 40 cases’ (Gibson, 2013: 7).

Many of the allegations concern British involvement in the Central Intelligence Agency’s (CIA’s) Rendition, Detention and Interrogation (RDI) programme, which saw a global network of secret prisons built and run by the CIA, wherein prisoners were held for months or years on end without access to legal representation or other contact with the outside world. Excruciating detail of this programme emerged in December 2014 with the publication of the redacted 499-page executive summary of the Senate Select Committee on Intelligence study (SSCI, 2014) (hereafter, SSCI report).^1^ This provided clear evidence of the CIA’s use of torture, including drowning to the point of unconsciousness, repeated beatings, the use of ice baths and hoses to induce hypothermia, sleep deprivation for more than a week at a time, painful stress positions for months at a time, prolonged confinement in extremely small boxes, and sexual assault by forced feeding through the rectum (SSCI, 2014: 3–4). Prisoners were threatened with power drills and subjected to mock executions, and were often beaten so severely that they passed out. Those detained in the CIA programme were subjected to a regime designed, as one interrogator stated, to take them ‘to the verge of death and back again’ (ICRC, 2007: 17).

Other allegations against British intelligence and security services involve abuses at Guantánamo Bay, other US military bases in conflict zones in Iraq and Afghanistan, and in detention facilities run by counterterrorism allies across Asia, the Middle East and Africa. Overall, the Detainee Inquiry identified ‘200 or so reported instances of the UK’s alleged involvement in, or awareness of, mistreatment of detainees’ (Gibson, 2013: 7), a figure that did not include allegations relating to military detention operations in Iraq and Afghanistan since these lay explicitly outside of the Inquiry’s scope (Cameron, 2010b: 1–2).

Some of these cases have led to civil action against the British government in UK courts. In return, the government has adopted a variety of measures to ensure either that key aspects of cases are heard in secret or that cases are ruled non-justiciable in their entirety. In some cases, the government has attempted to withhold the publication of key documents in open court, such as those which demonstrate that British intelligence knew about the torture of prisoners by the CIA before participating directly in their interrogation (e.g. UKCoA, 2010). Where UK courts have refused to accept government attempts to hold hearings *in camera*, such as in the case brought by five former Guantánamo Bay prisoners alleging British involvement in their unlawful imprisonment and treatment, the government has offered substantial payouts without any admission of liability on behalf of the British authorities (BBC, 2010; Casciani, 2012). The more recent passage of the Justice and Security Act in April 2013, with its introduction of so-called ‘closed material procedures’ into the main civil courts, was motivated largely by a desire to embed in law the executive’s ability to keep details regarding UK involvement in torture from reaching the public record. Similarly, in two related civil cases alleging British involvement — the rendition of Abdel Hakim Belhadj and Fatima Boucher to Libya (UKSC, 2014) and the rendition of Yunus Rahmatullah from Iraq to Afghanistan (UKSC, 2015) — the UK government has argued that either the foreign act of state doctrine or the doctrine of state immunity barred the courts from hearing the case.^2^

Relatedly, the UK government has undertaken a sustained attempt to ensure that details of British involvement in torture are not released in other forums. Government officials made regular representations to the Senate Committee to ensure that any mention of the UK was redacted from the SSCI report (Mason, 2014; UKFCO, 2014b). Likewise, in response to our own use of the Freedom of Information Act 2000, public authorities have engaged in a concerted effort to deny the release of information regarding UK involvement in torture. Authorities have deployed a range of techniques to deny release. For example, in refusing to provide information on the 20,000 documents gathered by the Detainee Inquiry, the Cabinet Office acknowledged that the information existed in their building but that it did not ‘hold’ the relevant information within the meaning of the Act (UKCO, 2014). Requests for information passed to the Detainee Inquiry have been denied as ‘vexatious’ (UKFCO, 2014a; UKMoD, 2014). Records of aircraft landings in Diego Garcia — a type of information freely provided in other contexts — have been withheld on public interest grounds given that US–UK bilateral relations ‘would be damaged’ if they were to be released (UKFCO, 2015a).^3^

Underlying these attempts to restrict the publication of evidence of involvement in torture is a consistent narrative of denial, articulated at the highest levels by the British government: Britain neither uses, condones nor facilitates torture or other cruel, inhuman or degrading treatment and punishment. Former Prime Minister Tony Blair has maintained that the UK responded to the Islamist terrorism threat with an unwavering commitment to human rights and international law. In a December 2005 press conference, he stated:

All I know is that we should keep within the law at all times, and the notion that I, or the Americans, or anybody else approve or condone torture, or ill treatment, or degrading treatment, that is completely and totally out of order in any set of circumstances. … I have absolutely no evidence to suggest that anything illegal has been happening here at all, and I am not going to start ordering inquiries into this, that and the next thing. (BBC, 2006)

More specifically, Blair testified that ‘British personnel neither assist nor are involved in rendition where there are grounds to believe that the person being rendered would face a real risk of torture or cruel, inhuman or degrading treatment’ (ISC, 2007: 17). This position was adopted by others in government, including Foreign Secretary Jack Straw. Speaking to the Foreign Affairs Select Committee in December 2005, Straw was clear:

Unless we all start to believe in conspiracy theories and that the officials are lying, that I am lying, that behind this there is some kind of secret state which is in league with some dark forces in the United States, and also let me say, we believe that Secretary Rice is lying, there simply is no truth in the claims that the United Kingdom has been involved in rendition full stop, because we have not been. (Straw, 2005)

This narrative of denial was supported by findings in two separate investigations by the body charged with holding the intelligence agencies accountable: the UK Parliament’s Intelligence and Security Committee (ISC). Both investigations examined the actions of the Secret Intelligence Service (SIS; hereafter MI6) and the Security Service (SyS; hereafter MI5), and heard testimony from the agencies that emphasised their adherence to the rule of law and human rights principles (ISC, 2005, 2007). Emphasis was placed on both the restraining effect on the Americans and the legality of the operations. MI5 testified that, while they may have had knowledge of particular rendition operations, there was no active assistance:

We were aware of people being moved from places: Afghanistan, Pakistan, Zambia and Gambia, usually to Guantánamo. But we are not party to the decision to do so, nor were we aware of routes or how it was done [redacted]. … Some of these renditions were dropped by the Americans after the Service had expressed concern at the proposal. (ISC, 2007: 50)

MI6, meanwhile, acknowledged that it had directly assisted ‘a very small number of renditions’ where prisoners were transferred back to their country of origin and where ‘we were certain that there was no risk of torture or cruel, inhumane and degrading treatment or torture (CIDT)’, and was clear that it had:

never assisted any renditions into so-called ‘black facilities’ … [nor] renditions to third countries, i.e. renditions to countries other than the USA or the detainee’s country of origin … [nor] renditions to the detainee’s country of origin where there was a real risk of CIDT, or which would breach the UK’s international obligations. (ISC, 2007: 51–52)

Likewise, in relation to ‘ghost prisoners’, held in secret locations without access to the International Committee of the Red Cross (ICRC), both agencies confirmed that ‘we do not know the locations or terms of their detention and do not have access to them’ (ISC, 2005: 20).

In both cases, the ISC concluded that UK intelligence agencies had only overseen minor, isolated infractions of UK policy, and overall had not acted improperly. There was, it stated, ‘no evidence that the UK Agencies were complicit in any “Extraordinary Rendition” operations’, where these involved a ‘real risk of torture or cruel, inhuman or degrading treatment’ (ISC, 2007: 29). On the question of passing intelligence on specific suspects to liaison partners, the ISC concluded that there were robust safeguards to ensure that British intelligence was not used in the torture or mistreatment of detainees:

Where there are concerns, the Agencies seek credible assurances that any action taken on the basis of intelligence provided by the UK Agencies would be humane and lawful. Where credible assurances cannot be obtained, the Chief of SIS explained ‘… then we cannot provide the information.’ (ISC, 2007: 13)

We argue in this article that denials by UK government officials, and the findings of the ISC in 2005 and 2007, are untenable. As a result of our work (alongside others), we are now in a position to argue that British involvement in torture during the ‘war on terror’ is a matter of historical record. Indeed, our analysis of the evidentiary material now in the public domain suggests that the UK has been deeply and directly implicated in abuse on a number of levels. British intelligence and security agencies have worked hand-in-glove with counterterrorism partners to identify and apprehend suspects and disappear them into secret detention where torture was endemic. Once suspects were in secret detention, British intelligence and security agencies have, in many cases, been intimately involved in the torture that took place, either by participating in the interrogations, by providing the intelligence that formed the basis of the torture or by receiving intelligence gained through torture. In addition, British territory has been used as a key logistical hub for the global network of secret detention and torture, with the UK facilitating the movement of suspects between secret prisons.

Before we examine this involvement in detail, in the next section, we account for the particular approach adopted by British intelligence and security agencies which ensured that the narrative of denial was more likely to be sustained.

## Understanding the British approach to torture in the ‘war on terror’

The use of torture by the British state is not new. Indeed, in many senses, US and UK practices of prisoner abuse since 2001 represented less a departure from the normative behaviour of leading liberal-democratic states as provided by a new ‘state of exception’, and more the re-emergence of practices that have lain at the heart of the US and British imperial projects for decades. The violence, including torture, that underpinned the expansion of European (including British) imperialism, as well as early US imperialism, is well documented (Bethell, 1984: 8–43; Elkins, 2005; Gallagher and Robinson, 1953; Killingray, 1973, 1986; Sorenson, 1968; Suret-Canale, 1971 [1964]; Tignor, 1976; Wood, 2003). Likewise, the use of torture by imperial states to defend their colonial gains against local forms of resistance has been discussed at length (Elkins, 2005; Vidal-Naquet, 1963), as has its use as an element of US global hegemony in the post-war era (Blakeley, 2009; Huggins, 1998; McCoy, 2006; Rejali, 2007; Valentine, 2000).

In the context of British imperial violence specifically, the use of torture was often brutal and widespread, occasionally reaching genocidal proportions. Moreover, British officials were often directly involved in the abuses meted out, as well as working alongside their colonial partners. Elkins (2005), for example, illustrates in detail how both British officials and colonial agents acting for the British state used torture widely in efforts to crush the Mau Mau insurgency in Kenya in the 1950s. Likewise, torture was used directly by British officials in attempts to forestall independence in Malaya (1948–1960), in Cyprus (1955–1959) and in Aden (1963–1967) (Curtis, 2003: 334–339). Moreover, within just a few years, similar practices were deployed much closer to home: in early 1971, the British government instigated the use of internment without trial in Northern Ireland to contain spiralling sectarian violence. This was accompanied by the development and routine use of the so-called ‘Five Techniques’: sleep deprivation, hooding, subjecting to noise, food and drink deprivation, and stress positions.

It is possible to trace similarities in the techniques of coercive interrogation used in Northern Ireland and elsewhere with techniques developed in the context of the ‘war on terror’. Indeed, it has become clear that the British colonial experience provided at least some of the inspiration, and legal justification, for the CIA’s ‘enhanced interrogation techniques’.^4^ However, notwithstanding these similarities, there remain significant differences between colonial-era and contemporary involvement in torture, each of which reflects the nature of the imperial projects of their time. Earlier involvement in torture formed part of a broader strategy of imperial policing and, as a result, could be blatant, widespread and extremely brutal, with British officials playing a direct role. Echoes of colonial-era torture can be seen in some of the abuses carried out by British forces in Iraq as the task of occupying restive populations led, at times, to direct involvement in prisoner abuse.^5^ Outside of the military occupation of Iraq, most of Britain’s involvement in prisoner abuse has taken on a neo-colonial form, with British agents acting alongside US partners and the security services of other sovereign states while simultaneously going to great lengths to ensure that they are never the detaining or responsible authority for the prisoners. In explaining this newly cautious approach, it is worth taking note of the domestic reaction among some Members of Parliament (MPs), elements of the press and the emerging human rights sector to the sheer brutality of colonial-era torture. The building controversy over the methods employed, particularly in Northern Ireland, where scrutiny was sharper, fed into the development of a network of contemporary policy and legal constraints on the use of violence by UK officials outside of military theatres of war. This manifested itself most obviously in the new guidelines issued by the Joint Intelligence Committee in June 1972. As authorised directly by Prime Minister Edward Heath, these guidelines made clear that the use of coercive interrogations by UK personnel would henceforth be banned. Subsequently reaffirmed in annual UK policy statements, this commitment remains a foundation stone in declaratory policy regarding the treatment of prisoners, and provides a clear backdrop against which UK agencies have had to operate in the post-9/11 era.

That the specific commitment to refrain from coercive interrogations was not rescinded following 9/11, and that the claim of exceptionalism did not lead to an overt suspension of international law as it pertains to the treatment of prisoners, marks out the UK response from that of its US counterpart. By February 2002, President Bush had publically declared that the Geneva Conventions did not apply to Taliban or al Qaeda prisoners, and that the US military (the CIA was notably absent from discussion) would treat those in its custody ‘in a manner consistent with the principles of Geneva’ as a matter of policy rather than law, and only to the extent ‘appropriate and consistent with military necessity’ (Bush, 2002: para. 3). Meanwhile, throughout 2002, the US Department of Justice, the White House and the CIA were, in secret, working together to redefine the interpretation of the prohibition against torture in US and international law in order to provide covert legal justification for a set of so-called ‘enhanced interrogation techniques’ (e.g. Bybee, 2002a, 2002b). In contrast, the British approach has been characterised by a ‘cautious pantomime of legal and procedural adherence’^6^ to international legal commitments and earlier policy statements, which has, in turn, conditioned and shaped the ways in which British personnel have been involved in torture.

In seeking to understand this approach, we need to examine how law and sovereign power have come to operate during the ‘war on terror’. In her compelling account, Judith Butler (2004: 51) argues that the suspension of law leads to a ‘new exercise of state sovereignty, one that not only takes place outside the law, but through an elaboration of administrative bureaucracies’. In the context of those detained in the ‘war on terror’, decisions regarding prisoner treatment are not taken by elected representatives or members of the judiciary, but rather by ‘petty sovereigns’; Butler (2004: 59) argues that ‘the power they wield to “deem” someone dangerous and constitute them effectively as such, is a sovereign power, a ghostly and forceful resurgence of sovereignty in the midst of governmentality’. As such, ‘the sovereignty produced through the suspension (or fabrication) of the rule of law, seeks to establish a rival form of political legitimacy, one with no structures of accountability built in’ (Butler, 2004: 66). In the circumstances of the ‘war on terror’, sovereignty has achieved this through the war prison, which has been used to manage specific populations, whether that be in Guantánamo, the numerous US detention facilities in Afghanistan and Iraq, or the secret CIA black sites. ‘Management’ of the war prison population, Butler (2004: 95–98) argues, is achieved not simply through suspending or fabricating law, but also through constituting the population ‘as less than human without entitlement to rights, as the humanly unrecognisable’.

In the context of the UK, here, the exercise of ‘petty sovereignty’ has not been enabled through the explicit suspension of previous legal and policy commitments under a ‘state of exception’; rather, it has been possible to operate outside the law, under the aegis of US exceptionalism, while simultaneously proclaiming to uphold international law in both spirit and letter. As such, ministers were able to declare that ‘national interests are served where human rights, democracy and the rule of law prevail’ (Straw, 2002) while, at the same time, developing mechanisms of procedural adherence to human rights commitments that nevertheless allowed plenty of wiggle room. Indeed, human rights commitments were restated repeatedly as part of a rhetoric of liberal foreign policy with a distinct normative flavour, which was particularly robust during the Labour government between 1997 and 2010 (e.g. Blair, 1999; Cook, 1997). This rhetorical, legal and policy ‘scaffold’, shaped both by adherence to Heath’s outlawing of torture and New Labour’s human rights rhetoric, provided the parameters within which UK intelligence services could both appear to be adhering to human rights standards and, at the same time, be party to the extreme abuse and torture of prisoners.

Procedurally, the UK intelligence and security agencies have been guided by a very particular approach, driven by two fundamental principles. First, British agencies were, at all times, to avoid the formal legal custody of prisoners. Although actively involved in capture and detention operations, the UK agencies were very rarely the detaining power, with all involvement taking place within the context of supporting partner operations. Second, British agencies were, at all times, to avoid the direct abuse of prisoners. Participation in detention, rendition and interrogation operations where abuse was known to be taking place, or where it was common sense to assume that abuse would take place, was deemed legitimate by British intelligence and security officials. Adhering to these principles ensured that the UK could remain full counterterrorism partners of the US and other allies. It could target those considered threats to national security by ensuring that they were subjected to harsh treatment on the dubious assumption that they would elicit valuable intelligence. At the same time, the UK could insulate itself from allegations of abuse, with both the intelligence and security agencies themselves, and ministers with responsibility for the agencies, able to maintain their narrative of denial, and to continue to insist that the UK counterterrorism effort was underpinned by a robust commitment to human rights.

This particular approach emerged immediately after the declaration of a ‘war on terror’ by the US, and the start of military operations in Afghanistan. As British intelligence on the ground in Afghanistan began to interrogate those held by counterterrorism partners, guidance was issued by headquarters in London. Access to prisoners in Afghan detention facilities was permitted by MI6 providing that two strict conditions were adhered to: ‘a) that at no time were they to be under SIS control “as this would mean that we would incur Geneva Convention responsibilities for them”, and b) that they “would not be subject to coercion or torture and generally treated humanely”’ (Gibson, 2013: 47). When MI6 and MI5 were given permission by the Americans to interrogate US-held prisoners in Afghanistan, in early January 2002, it quickly became clear to British agents that prisoner mistreatment was occurring. One MI6 officer who conducted an interrogation of a US-held prisoner reported to London on 10 January 2002 ‘observations on the circumstances of the handling of [the] detainee by the US military before the beginning of the interview’ (ISC, 2005: 13). The details of this report remain classified, although the reply by headquarters, copied to all MI5 and MI6 officers in Afghanistan, admitted that prisoners ‘may not be being treated in accordance with the appropriate standards’:

With regard to the status of the prisoners, under the various Geneva Conventions and protocols all prisoners, however they are described, are entitled to the same levels of protection. You have commented on their treatment. It appears from your description that they may not be being treated in accordance with the appropriate standards. Given that they are not within our custody or control, the law does not require you to intervene to prevent this. That said, HMG’s [Her Majesty’s Government] stated commitment to human rights makes it important that the Americans understand that we cannot be party to such ill treatment nor can we be seen to condone it. In no case should they be coerced during or in conjunction with an SIS interview of them. If circumstances allow, you should consider drawing this to the attention of a suitably senior US official locally. It is important that you do not engage in any activity yourself that involves inhuman or degrading treatment of prisoners. (ISC, 2005: 13–14)

That the MI6 officer witnessed abuse in a US facility in Afghanistan in early 2002 is unsurprising. Human Rights Watch found that the mistreatment of prisoners in the largest detention site, part of Bagram Airbase, was ‘especially harsh’ in the first months after its opening. Prisoners reported being subjected to continuous bright lights, sleep deprivation and shackling in painful stress positions for weeks on end, as well as physical abuse during interrogations (HRW, 2004). Those held at Kandahar Airbase in early 2002, meanwhile, reported being beaten so badly that they suffered broken bones or were unconscious, with others forced to lie on the frozen ground outside until they were numb with cold. Allegations of summary executions by US forces undertaking detention operations also exist (HRW, 2004: 38–39). In the context of significant prisoner abuse in US facilities in Afghanistan, therefore, UK officials were explicitly advised to ensure only that mistreatment did not take place ‘during or in conjunction with’ British interrogations, and that only ‘if circumstances allow’ should they bring the mistreatment of prisoners to the attention of partner agencies. Perhaps unsurprisingly, the Detainee Inquiry found a number of cases where British intelligence was aware of specific mistreatment by its partners but did not report this (Gibson, 2013: 23). In other cases, record keeping by officers on the ground was so poor that there were no interrogation records or records with no or limited details of the welfare of the prisoner concerned (Gibson, 2013: 29). This memo exemplifies the bureaucratic role played by headquarters in creating the appearance of adherence to human rights standards while simultaneously making room for the expansion of petty sovereignty on the ground in Afghanistan and elsewhere.

As we examine the contours of British torture in the next section, we will see procedural compliance playing out at many points: in cases where British agents absented themselves from the interrogation room when torture occurs; in cases where British Special Forces were permitted to detain but not interrogate; in cases where detention was authorised but formal arrest was not; and in cases where intelligence was passed to the CIA and other parties to inform interrogations conducted through torture, and subsequently received from the torture chambers. The role of British officials as petty sovereigns is also apparent in the polite emails exchanged between MI6 personnel and their Libyan counterparts. Torture and the illegality of the UK-orchestrated kidnappings and subsequent interrogations are alluded to in clean terms, with petty squabbles over whether UK or US officials should be the primary beneficiaries of the intelligence product seeming to matter more to the British correspondent than the serious ways in which national and international law had just been flouted. This very clearly illustrates the bureaucratic role that British parties to prisoner abuse end up playing, almost completely disconnected from the serious matters of justice, even life and death, that are actually at stake.

## The contours of British torture in the ‘war on terror’

As we have seen, the approach adopted by the UK left a great deal of latitude for involvement in torture and other prisoner abuse, as long as officials continued only to play a supporting role to that abuse, and as long as it did not take place in a way that would leave officials unable to deny involvement. As we will now show, by outlining the ways in which British agencies were involved in prisoner abuses, this freedom of movement was fully exploited by the UK during the ‘war on terror’.

### Direct support for prisoner capture and transfer to secret detention and torture

UK intelligence and security agencies have played a key role in identifying and locating terror suspects, apprehending them, and transferring them for detention and interrogation under torture by allies. The British role was either to supply the intelligence needed for the apprehension or to take part in capture operations as formal secondary partners, ensuring that they were not directly responsible for prisoners.

British intelligence agencies were heavily involved in the CIA’s rendition programme, providing intelligence leading to the capture of suspects, or providing direct logistical assistance in rendition operations. In the case of Bisher al-Rawi and Jamil el-Banna, the passing of UK intelligence to the CIA about their whereabouts was central to their rendition to Afghanistan, where they were held in secret CIA detention before transfer to US military custody. Both men had been detained in the UK in early November 2002, several days before their disappearance, with MI5 providing to the CIA details of the men’s detention and their travel plans to The Gambia (MI5, 2002). This led directly to their arrest in Banjul, their transfer into the CIA programme and their disappearance for several weeks before emerging as US military prisoners at Guantánamo Bay.^7^ The ISC report exonerated British intelligence from responsibility for the mistreatment of al-Rawi and el-Banna, pointing to a caveat placed on the memo to the CIA prohibiting executive action on the basis of the intelligence supplied, and concluding that ‘we accept that the Security Service did not intend the men to be arrested’ (ISC, 2007: 40). However, placed within the context of numerous cases where the UK worked to deliver suspects to secret detention, it is now clear that this conclusion by the ISC is either untenable or relevant only to this case.

Indeed, documents gathered by the Detainee Inquiry make clear that UK involvement in rendition was widespread. Although ministerial approval was granted in a number of these cases, the appropriateness of such involvement by UK intelligence was considered by the Inquiry to ‘be open to question’. In other cases, it was not clear that ministerial approval was either sought or granted (Gibson, 2013: 34). Moreover, crucially, the classified documents reviewed by the Inquiry outlined numerous instances where UK agencies were involved in renditions to torture, where the likelihood or certainty of torture was clear:

In a number of instances where the documents indicate that there was some level of UK approval for, or assistance in, a rendition operation by a third country, or the feeding in of questions afterwards, the renditions or proposed renditions were to countries where there were objective grounds for concern about the receiving country’s standards of detainee treatment. In some cases, the UK Courts would not at the time have permitted the UK to deport suspected extremists to those countries because of the risk of mistreatment contrary to Article 3 of the European Convention on Human Rights. (Gibson, 2013: 36)

In other words, UK intelligence agencies played an active role in arranging the rendition of suspects to third countries, outside of any legal process, where the risk of torture was high. Although the specific details of most of these cases remain classified, the central involvement of MI6 in the rendition of Libyan dissidents and their families to Gaddafi’s Libya has recently been exposed. Documents obtained by Human Rights Watch from a government building in Tripoli in September 2011, in the immediate aftermath of the fall of the Gaddafi regime, provide compelling evidence of British involvement in these operations (HRW, 2012). In one such operation, dissident Sami al-Saadi and his family, including his four children, were rendered from Hong Kong to Libya in March 2004. One memo from the CIA to its Libyan counterpart, dated 23 March 2004, was clear that they were ‘aware that your service had been cooperating with the British to effect [al-Saadi’s] removal to Tripoli’, and offered to step in to ‘render [him] and his family into your custody’ (CIA, 2004a). Once in Libya, al-Saadi was detained for six years, during which time he was subjected to beatings with ropes and sticks, as well as electric shocks to the neck, chest and arms.

In a similar operation, dissident Abdel Hakim Belhadj (also known as Abu Abdullah al-Sadiq) was rendered with his wife, Fatima Bouchar (who was pregnant at the time), from Malaysia to Libya. MI6 were aware of their initial detention in Malaysia, and took an active role in organising their rendition back to Libya (MI6, 2004a). The CIA were also made aware, and took the lead in organising their rendition back to Libya (via a refuelling stop in Diego Garcia) (CIA, 2004b, 2004c, 2004d, 2004e). That Britain played a key role in this rendition operation was confirmed by a memo from Mark Allen, then Director of Counterterrorism at MI6. Sent to his counterpart in Libya, Musa Kusa, the memo explicitly congratulates Kusa on the ‘safe arrival’ of Belhadj and discusses securing direct British access to the detainee’s interrogations:

Most importantly, I congratulate you on the safe arrival of Abu Abd Allah Sadiq [Belhadj]. This was the least we could do for you and for Libya to demonstrate the remarkable relationship we have built over the years. I am so glad. I was grateful to you for helping the officer we sent out last week. Abu ‘Abd Allah’s information on the situation in this country is of urgent importance to us. Amusingly, we got a request from the Americans to channel requests for information from Abu ‘Abd Allah through the Americans. I have no intention of doing any such thing. The intelligence on Abu ‘Abd Allah was British. I know I did not pay for the air cargo. But I feel I have the right to deal with you direct on this and am very grateful for the help you are giving us. (MI6, 2004b)

The documents now in the public domain provide a unique window into the role played by MI6 in the rendition of these Libyans. However, as the Detainee Inquiry makes clear, this was no exception, and UK intelligence was involved in numerous similar operations alongside the CIA as part of its RDI programme, albeit as supporting partners.

The role played by the UK in this programme is highlighted by the degree to which British territory was used by CIA aircraft as refuelling stops while undertaking rendition operations. Collation and analysis of flight data associated with CIA rendition aircraft, and the correlation of this with data concerning prisoner transfers within the RDI programme, has allowed us to establish that UK involvement in the rendition programme was much more extensive than previously thought (Cobain, 2013a; Raphael and Blakeley, 2013). British territory was used for the rendition of at least 24 prisoners between secret prisons, some of whom were subjected to torture. These include the two prisoners acknowledged to have passed through Diego Garcia in 2002 (Milliband, 2008), as well as Belhadj in March 2004. Mainland UK was used in the rendition of so-called ‘high-value detainees’ to secret detention in Poland, including Abu Zubaydah, Abd al-Rahim al-Nashiri, Ramzi bin al-Shibh and Khaled Sheikh Mohammed, all of whom were tortured at the site (Raphael et al., 2015; SSCI, 2014). Others were taken to secret CIA prisons in Afghanistan (Zubair, Ibn Sheikh al-Libi, Sanad al-Kazimi, Saleh Qaru), Romania (Abu Faraj al-Libi, Abu Munthir al-Maghrebi, Janat Gul) and Lithuania. Still more were rendered to proxy detention in Egypt, Jordan or Morocco on aircraft that used UK territory as a staging post.^8^ The UK government has consistently refused to investigate these flights, and has even claimed that it would be impossible to do so (UKFCO, 2015b; ISC, 2007: 57–62). However, given that we now know that the same aircraft were used in operations with direct British involvement, there appears to be a *prima facie* case for assuming that British intelligence were aware of these additional landings in the UK. Indeed, evidence of UK complicity in this regard has been considered robust enough to launch and sustain a police investigation into the matter (BBC, 2013).

UK Special Forces also played a key role in operations to capture terror suspects and transfer them to secret detention and torture, again while ensuring that the British hand remained hidden. In Iraq, Special Forces worked closely with their US counterparts to locate and apprehend high-value targets from the Saddam regime and al Qaeda, and to transfer them into a network of secret detention facilities. Task Force 121, later renamed Task Force 6-26, was a key US interagency unit comprising US Army, Navy and Air Force Special Forces and the CIA’s paramilitary wing, the Special Activities Division, which led on hunting down, secretly detaining and interrogating suspects. Detentions took place across Iraq, often in nondescript buildings in cities including Falluja, Balad, Ramadi and Kirkuk, with large backyards for helicopter drop-offs and pick-ups (Schmitt and Marshall, 2006). Camp Nama was central: a secret site next to Baghdad International Airport where torture chambers used by the Saddam regime were converted into interrogation rooms. Torture and abuse were rife, with suspects held in cells the size of dog kennels, routinely hooded, severely beaten and subjected to electric shocks. In some reports, prisoners were used as targets in paintball games (Schmitt and Marshall, 2006).

UK personnel were deeply involved in the operation of this secret prison and torture network. Royal Air Force (RAF) and Army Air Corps squadrons helped with guard and transport duties at Nama, guarding prisoners held in cells that were, according to one British ex-serviceman, ‘made of wire mesh with sloping corrugated roofs. They were chest high, and two feet wide. There were about 100 of them, in three rows, and they always appeared to have at least one prisoner in each’ (Cobain, 2013b). Many of the prisoners were apprehended by ‘snatch squads’ consisting of UK personnel working alongside US counterparts. In some cases, units were made up almost exclusively from UK Special Forces, accompanied by a lone US officer to record the capture and ensure that the operation (and therefore subsequent detention) was badged as American. As one British serviceman who served at Nama recalled:

The Americans went out to bring in prisoners every night, and British Special Forces would go out once or twice a week, almost always with one American accompanying them. The prisoners would be brought in by helicopter, usually one at a time, although I once saw five being led off a Chinook. They were taken into a large hangar to be bagged and tagged, a bag put over their heads and their hands plasticuffed behind their backs. Then they would be lifted or thrown on to the back of a pick-up truck and driven to the Joint Operations Centre [where much of the interrogation and torture took place]. (Cobain, 2013b)

Alternatively, UK forces would detain suspects without effecting a formal arrest. A former member of the Special Forces explained that:

As UK soldiers within this Task Force a policy that we would detain individuals but not arrest them was continually enforced. Since it was commonly assumed by my colleagues that anyone we detained would subsequently be tortured this policy of detention and not arrest was regarded as a clumsy legal tool used to distance British soldiers from the whole process. (Statement from Ben Griffin, 25 February 2008, quoted in Cobain, 2013b)

UK officers were generally not provided access to interrogation rooms, and avoided being present during the abuse. When they were directly involved, this was clearly in contravention of the guidance issued to ensure that the UK involvement remained ‘clean’. One US interrogator, speaking to Human Rights Watch, made this clear in regards to abuse that he witnessed by a member of the Special Air Service (SAS) at Nama:

[It] was a beating in a kind of a bunker behind the main facility … this British guy actually who wasn’t supposed to be interrogating anybody … But we went back there and he gave the guy a pretty good pounding. Nothing really in the face. A lot of stomach shots, and I would say two or three groin shots, very harsh. A knee to the abdomen. Thrown against the wall and so forth … he [the British soldier] decided to go that route and get physical with him … [W]e ended up cutting in … I took the prisoner back and my partner took the other guy. It was reported. They weren’t upset about any type of abuse or anything. They were just upset that he [the British soldier] was interrogating anybody at all, because it was not in adherence with the rules. Because he wasn’t American or he wasn’t, you know, signed on to do that type of job. (HRW, 2006: 11–12)

Accusations of British torture at Camp Nama have also been made by Yunus Rahmatullah, a Pakistani businessman captured in Iraq in February 2004. Court documents filed on behalf of Rahmatullah allege that he was captured by the joint UK–US Special Forces Task Force, and subjected to torture by both UK and US soldiers, including the use of dogs, waterboarding and confinement in a box just 40cm high (Finn and Raphael, 2014; UKHC, 2014). That the British held Rahmatullah was finally acknowledged by the UK government in 2009, as was his eventual transfer to US forces, who rendered him to Afghanistan and detained him without charge for over 10 years (Hutton, 2009).

### Direct support for interrogation under torture

As British actions in Camp Nama show, there is evidence that UK intelligence and security agencies not only were involved in the capture and transfer of suspects, but also provided on-the-ground support for, or even took the lead in, prisoner mistreatment. The direct involvement of British personnel in abuse appears to be limited to the context of Special Forces operations in military theatres. In other locations around the world, UK intelligence agencies appear to have maintained a safe distance from the torture taking place. Nonetheless, the evidence of British involvement in interrogations under torture is compelling.

The Detainee Inquiry found that UK intelligence officers were aware of ‘a range of treatment issues’ by liaison partners. These ‘issues’ included hooding, stress positions, sleep deprivation, physical assaults, inhumane detention facilities and ‘questionable methods of transfer between detention sites’. In some cases, these issues did not affect British involvement in the mistreatment. As the Inquiry noted: ‘there are some instances where UK officers continued to engage with detainees held by liaison partners in various locations after ill-treatment had either been witnessed or alleged’ (Gibson, 2013: 24).

There are numerous examples of UK intelligence providing direct support for interrogations under torture. MI5 was involved in the case of British resident Binyam Mohamed, detained in Pakistan in April 2002, and tortured while held incommunicado in an Inter-Services Intelligence (ISI) interrogation centre. Mohamed was ‘hung up for a week by a leather strap around the wrists so he could only just stand’. At one point, a Pakistani agent loaded a semi-automatic gun:

He pressed it against my chest. He just stood there. I knew I was going to die. He stood like that for five minutes. I looked into his eyes, and I saw my own fear reflected there. I had time to think about it. Maybe he will pull the trigger and I will not die, but be paralyzed. (Testimony cited in Reprieve, 2008: 6)

While in Pakistan, Binyam Mohamed was visited by an officer from MI5 (who was referred to as Witness B in subsequent litigation). Before the officer left London, MI5 was briefed on Mohamed’s mistreatment by the CIA, and clearly knew that he was being tortured. A subsequent court case revealed that the CIA had passed at least 42 documents to MI5 before its officer travelled to Pakistan. These documents showed that Mohamed had been interrogated by US authorities while in Pakistani custody, during which he had been subjected to continuous sleep deprivation, shackling and threats of being ‘disappeared’. The documents also showed that this mistreatment was ‘having a marked effect upon him and causing him significant mental stress and suffering’, and that, as a consequence, he was being kept under self-harm observation. The UK High Court concluded that ‘the reports provided to the SyS made clear to anyone reading them that BM [Binyam Mohamed] was being subjected to the treatment that we have described and the effect upon him of that intentional treatment’, and that this treatment ‘could readily be contended to be at the very least cruel, inhuman and degrading treatment by the United States authorities’. The Court concluded that the mistreatment reported by the CIA to MI5, if it ‘had been administered on behalf of the United Kingdom, would clearly have been in breach of [the UK’s policy commitments]’ (UKCoA, 2010, paras 32, x).

The Detainee Inquiry also found that prisoners were exposed to ‘harsh’ sessions involving abuse from foreign security forces, and ‘softer’ sessions with British personnel (Gibson, 2013: 23). In the context of the US–UK partnership, the Inquiry found that there was ‘collaborat[ion] over their approach and conduct during detainee interview sessions’, with UK intelligence adopting ‘a more reassuring and friendly manner, which contrasted with the manner adopted by liaison partners’. This good cop/bad cop strategy appeared to the Inquiry at first glance to be intimately connected with detainee abuse, and it explicitly questioned whether, ‘in some cases, UK officers may have turned a blind eye to the use of specific, inappropriate techniques or threats used by others and used this to their advantage when resuming an interview session with a now compliant detainee’ (Gibson, 2013: 28).

Such a ‘good cop/bad cop’ strategy has been reported in several contexts, especially where British residents were detained by partner intelligence agencies on the UK’s request. In one case, Jamil Rahman, a British-Bangladesh dual national, was detained for more than two years by Bangladeshi intelligence officers, supported by UK officials. According to Rahman, two men from MI5 were involved in his interrogation, but would leave the room when he refused to answer their questions. While absent, he would be severely beaten before they returned to resume the interrogation (Cobain, 2009). Similar allegations have been made by British-Pakistani dual nationals held in Pakistan. Research by Human Rights Watch found ‘no evidence of UK officials directly participating in torture’, yet concluded that:

UK officials engaged in acts that virtually required that they knew about the use of torture in specific cases. In some cases [British officials met detainees] shortly after sessions in which the individuals had been tortured, when it was likely that clear and visible signs of torture were present. (HRW, 2009: 2)

In one case, UK citizen Salahuddin Amin was detained by the ISI throughout 2004 and tortured repeatedly, including with an electric drill. Between torture sessions, Amin was repeatedly driven to another location where he says that he was questioned by men who identified themselves as MI5 officers. These men asked him the same questions as during the torture sessions, or further questions that then became the basis for future torture sessions (HRW, 2009: 21–22).

Even where British intelligence officers did not have direct access to prisoners, there have been numerous occasions where the UK has passed questions to counterterrorism allies for use during interrogations, and has received intelligence reports generated as a result of such interrogations. This has been the case even where the likelihood of abuse has been high. Indeed, the ISC was told of ‘a number of cases where individuals detained by foreign liaison services have provided, directly or indirectly, important intelligence that has helped to prevent attacks on the UK’. This included intelligence provided by Khaled Sheikh Mohammed during 2003, while in ‘detention … place unknown’, including names of six al Qaeda suspects targeting the UK (ISC, 2007: 12). Mohammed was, at that time, being held and tortured in a CIA black site in Poland, where he was subjected to standing sleep deprivation, beatings, kneeling stress positions and 15 separate waterboarding sessions, comprising over 180 applications of the waterboard. The SSCI report details how, during the torture sessions, Mohammed provided information on plots against Heathrow Airport and Canary Wharf, which was then passed to counterterrorism partners (SSCI, 2014: 83–93, 300).

In the case of Binyam Mohamed, MI5 continued to receive reports from CIA interrogations at later stages of his secret detention. As a result of these reports, UK intelligence determined that ‘BM might have further relevant information to provide and that it was in the interests of the national security of the United Kingdom to seek his responses to further questions, ideally through a further interview conducted by the SyS’ (UKCoA, 2010: para. 29). MI5 was not granted access to Mohamed by the CIA, although they were aware that Mohamed was being held ‘in a covert location where he was being debriefed’. As a result, they began channelling questions through the CIA for use in interrogations (UKCoA, 2010: para. 35A). In one telegram, dated 25 October 2002, MI5 declared itself ‘grateful for the opportunity to provide material to be used in the current debriefing’. Further questions were sent on 5 November 2002, and MI5 received multiple reports from the interrogations in Morocco in February 2003. These reports led MI5 to again request direct access to Mohamed, and to send over 70 further questions (UKCoA, 2010: para. 30). Throughout late 2002 and early 2003, as UK intelligence were supplying questions for, and receiving reports from, Mohamed’s interrogations, Mohamed was being brutally tortured by his captors, who (among other abuses) repeatedly cut his genitals with a scalpel (Reprieve, 2008: 12).

It appears that UK intelligence remained deeply involved in the interrogation under torture of Abdel Hakim Belhadj and Sami al-Saadi after arranging their rendition to Libya. Allen’s memo to Kusa (discussed earlier) made clear that the UK expected direct access to these suspects once in Libyan custody (MI6, 2004b). Belhadj has testified that he was interrogated by three British intelligence officers during two sessions, each lasting around two hours (Sengupta, 2011). UK intelligence agencies sent over 1600 questions to their Libyan counterparts for the interrogations of Belhadj and al-Saadi (Cobain, 2015; Taher and Rose, 2012). Likewise, Sami al-Saadi says that he was interrogated by Libyan, American and British agents while being detained and tortured in Libya.

## Conclusion

We have shown that despite repeatedly denying its involvement in prisoner abuse, and repeatedly claiming that the UK government unreservedly condemns torture, since 9/11, the UK has been deeply and directly involved in torture. We have provided the most detailed and in-depth account to date of this involvement by compiling and analysing a range of relevant evidentiary material. We have also argued that there has been a peculiarly British approach to torture in the ‘war on terror’ that has lent itself very well to sustaining a narrative of denial.

To explain this peculiarly British approach, we have drawn on Butler’s work to argue that it can be best understood within the context of how law and sovereign power have come to operate during the ‘war on terror’. We have shown that by invoking a state of emergency to justify the suspension of law, the way was paved for a new exercise of state sovereignty, not by elected state representatives or the judiciary, but by unelected officials, both within the US and also much further afield. Without any mechanisms for legal challenge or review, officials could take the decisions on who should be arbitrarily detained and subjected to all kinds of abuses. New forms of sovereignty manifested themselves in the management of specific suspect populations, through the war prison, whether in Guantánamo Bay or the secret CIA black sites dotted across the world, allocating power to a growing number of executive and administrative officials who have no legitimacy to determine the life or death or access to justice of those they detain. This included British intelligence officials.

We have shown that there was no need for UK authorities to invoke a state of emergency. Indeed, the UK government could continue to insist on its compliance with international human rights standards and its ongoing commitment to the prohibition of torture by UK agents. The state of exception had been established by the US, and was, indeed, beyond the UK’s control. Arbitrary detention and torture were, in that sense, a done deal. All that Britain needed to do to benefit from this extension of sovereign power was to maintain the appearance of adherence to international law, and unelected officials found various ways to do this. Meanwhile, by not committing UK agents to the principles underpinning international law that prohibits torture and prisoner abuse, huge latitude was given to officials to act as ‘petty sovereigns’. We see this in the slippery language around UK obligations under the Geneva Conventions, in the pantomime of British agents leaving the room when torture took place and in the petty correspondence between British agents and Libyan counterparts in which an MI6 official seeks to assert himself as being at the top of the pecking order when it came to intelligence sharing by the Libyans, while entirely sidestepping the horrors of how such intelligence was obtained.

What our findings illustrate is how instrumentally law has been used through both its suspension and its partial application. With Butler, we share the view that law has a very important role to play ‘in the articulation of an international conception of rights and obligations that limit and condition claims of state sovereignty’ (Butler, 2004: 98). There are necessarily limits to freedom: coercive actions must sometimes be taken against individuals to protect communities from harm. However, as Conor Gearty (2013: 114–115) persuasively argues, ‘human rights and the rule of law return to guide us to the right way to tackle this dilemma’. They will continue to be extremely important instruments in holding to account elected and non-elected officials where they illegitimately allocate sovereign power to themselves. Indeed, they are essential if we are to challenge new forms of sovereignty that are halting the expansionist trend of universal human rights and freedoms that began in the aftermath of the Second World War, and that would put at risk the security and freedom of all citizens.

## Notes

1. The full report, some 6700 pages long, remains classified.

2. Such arguments reached the Supreme Court in November 2015, which, at the time of writing, has yet to issue its judgement.

3. Our experience reflects that of others who have attempted to use the Freedom of Information Act in the context of UK involvement in torture, for example, the efforts of the All Party Parliamentary Group (APPG) on Extraordinary Rendition (see: http://www.extraordinaryrendition.org/).

4. One of the architects of the CIA’s torture programme, Assistant Attorney General Jay S. Bybee, explicitly referred to the use of these techniques in Northern Ireland when providing legal justification for torture in the ‘war on terror’ (Bybee, 2002a). Similarities in practice followed this authorisation, although the question of how torture techniques are transmitted historically, geographically and jurisdictionally is complex (see Rejali, 2007). Nonetheless, it may be possible to trace connections in this context. Stress positions used in Northern Ireland, such as forced standing for hours and days on end, were deployed later by the CIA, although to even greater extremes. Likewise, methods used to disorient, humiliate and intimidate prisoners in the RDI programme bear striking resemblance to practices established in Northern Ireland. The use of hooding, shackling, sensory deprivation, intimate physical examinations for no obvious medical or security reason, and methods to disguise the location of detention sites characterise both cases (Cobain, 2012: 141–142; PACE, 2006: paras 84–87). Mock drowning, similar to the use of water torture by the CIA, was employed at police stations in Belfast. In many cases, and in a clear parallel to ‘war on terror’ torture, interrogators and guards often went beyond that which had been authorised. Prisoners were beaten or burned with cigarette lighters, and threatened with more severe torture or even murder (Cobain, 2010).

5. See, for example, the case of the abuse and death in custody of Iraqi national Baha Mousa (Gage, 2011; Williams, 2012).

6. We thank one of the anonymous reviewers for this phrase.

7. For full details of the men’s treatment as part of the CIA programme, see: http://www.therenditionproject.org.uk/prisoners/rawi_banna.html

8. A full list can be generated from our Rendition Flights Database search tool, and typing ‘UK’ into the ‘Circuit Landed In’ search box, see: http://www.therenditionproject.org.uk/flights/renditions/index.html
